# Inflammasome-Independent Modulation of Cytokine Response by Autophagy in Human Cells

**DOI:** 10.1371/journal.pone.0018666

**Published:** 2011-04-07

**Authors:** Tania O. Crişan, Theo S. Plantinga, Frank L. van de Veerdonk, Marius F. Farcaş, Monique Stoffels, Bart-Jan Kullberg, Jos W. M. van der Meer, Leo A. B. Joosten, Mihai G. Netea

**Affiliations:** 1 Department of Medicine, Radboud University Nijmegen Medical Center, Nijmegen, The Netherlands; 2 Nijmegen Institute for Infection, Inflammation and Immunity (N4i), Radboud University Nijmegen Medical Center, Nijmegen, The Netherlands; University of California Los Angeles, United States of America

## Abstract

Autophagy is a cell housekeeping mechanism that has recently received attention in relation to its effects on the immune response. Genetic studies have identified candidate loci for Crohn's disease susceptibility among autophagy genes, while experiments in murine macrophages from *ATG16L1* deficient mice have shown that disruption of autophagy increases processing of IL-1β and IL-18 through an inflammasome-dependent manner. Using complementary approaches either inducing or inhibiting autophagy, we describe modulatory effects of autophagy on proinflammatory cytokine production in human cells. Inhibition of basal autophagy in human peripheral blood mononuclear cells (PBMCs) significantly enhances IL-1β after stimulation with TLR2 or TLR4 ligands, while at the same time reducing the production of TNFα. In line with this, induction of autophagy by starvation inhibited IL-1β production. These effects of autophagy were not exerted at the processing step, as inflammasome activation was not influenced. In contrast, the effect of autophagy on cytokine production was on transcription level, and possibly involving the inhibition of p38 mitogen activated protein kinase (MAPK) phosphorylation. In conclusion, autophagy modulates the secretion of proinflammatory cytokines in human cells through an inflammasome-independent pathway, and this is a novel mechanism that may be targeted in inflammatory diseases.

## Introduction

Autophagy is a conserved mechanism for degradation of defective organelles and long-lived proteins, that plays an important role in the homeostasis of the cell by recycling cytoplasmic cargo for aminoacid and energy re-use [1]. Autophagy comprises three main processes – chaperone-mediated autophagy, microautophagy and macroautophagy. The latter, henceforth referred to as autophagy, is characterized by the sequestration of cytosolic proteins and organelles into double-membrane vesicles called autophagosomes. These autophagosomes maturate through fusion with lysosomes, a process that will eventually lead to the breakdown of the protein content [2]. Through these effects, autophagy has been demonstrated to be a biological response of the cell in stressful situations, traditionally during starvation and growth factor deprivation, ensuring the degradation of old structures with the purpose of sustaining the essential anabolic processes of the cell [3]. Consequently, through its main roles in survival and housekeeping, the process of autophagy has gained relevance in the context of human pathologies like neurodegenerative diseases [4], cancer [5], lysosomal diseases [6], and ageing [1].

In addition to its role in cell survival, autophagy is emerging as a process of high importance for the host defense, influencing both the innate and adaptive immune responses [7]. This role is exerted at three levels [8]: direct involvement in engulfment and removal of intracellular pathogens [9], [10], facilitation of the MHC class II antigen presentation [11], and support of the T-lymphocyte development and survival for optimal protective immune responses [12], [13]. Whether the involvement of autophagy in the immune processes is due to the autophagic mechanism itself or to independent effects of autophagy-related genes [14], is still a matter of debate.

Genetic studies [15]–[17] have identified allelic variants of the autophagy genes *ATG16L1* (autophagy related 16-like 1) and *IRGM* (immunity related GTP-ases, M) as important risk factors for Crohn's disease, an autoinflammatory disease characterized by severe chronic inflammation of the gut mucosa [18], [19]. One possible explanation for the involvement of defective autophagy in Crohn's disease inflammation couples the risk alleles in *ATG16L1* and *NOD2* (nucleotide-binding oligomerization domain containing 2) to an impaired clearance of microorganisms [20], [21]. The persistence of bacteria in the mucosa could induce an inflammatory reaction, leading to the clinical features of Crohn's disease. An alternative explanation has also been proposed by Saitoh *et al.*, who demonstrated that the disruption of autophagy in *ATG16L1*-deficient murine macrophages enhances the LPS-induced IL-1β production through an inflammasome-dependent pathway [22]. However, whether autophagy has similar inhibitory effects in human cells is not known.

In the present study, we assessed the effect of autophagy on the production of proinflammatory cytokines in human cells. By stimulating human peripheral blood mononuclear cells (PBMCs), we show that the inhibition of autophagy increases IL-1β production after stimulation with TLR2 or TLR4 ligands. These effects were specific for the modulation of IL-1β secretion, while TNFα production was significantly reduced by agents that inhibited autophagy. In contrast to murine macrophages, these effects on human cells were exerted at the transcriptional level, rather than at the level of the inflammasome.

## Results

### Western blot assessment of autophagy marker LC3-II shows that starvation induces, whereas 3MA treatment inhibits, the autophagic process

**Figure 1:**
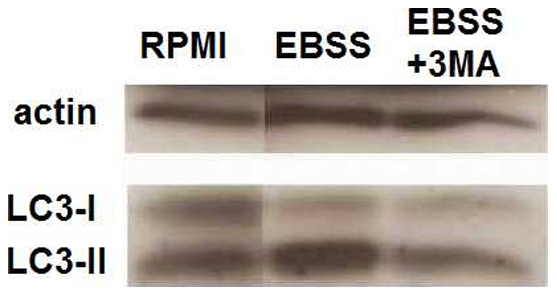
Assessment of LC3 I and II levels in PBMCs under starving conditions or pharmacological treatment with 3MA. Human PBMCs were pre-incubated for 1 hour at 37°C in either RPMI, starvation medium (EBSS) or EBSS with 3MA (10 mM) in which inhibitors of lysosomal fusion have been added: Ammonium chloride 20 mM and Leupeptine 100 µM. This was followed by 3 hours stimulation with culture medium, LPS (10 ng/ml) or Pam3Cys (10 µg/ml) prepared in the corresponding media (RPMI or EBSS). Cells were lysed and western blot of LC3 fractions I and II has been performed.

Microtubule associated protein 1 light chain 3 (LC3) is one of the autophagy-related proteins involved in the direct formation of the autophagosome. Through conjugation with phosphatidylethanolamine, the cytosolic LC3-I is transformed to LC3-II which is then bound to the autophagosome membrane, being indicative of autophagic activity inside the cell [23]. In order to verify the modulation of autophagy using the typical induction method by starvation and the inhibition method using the pharmacological inhibitor 3-Methyl Adenine (3MA) in the human PBMCs system used in this study, Western blotting of the two LC3 fractions was performed. Freshly isolated human PBMCs treated with the starvation medium EBSS (Earle's Balanced Salt Solution) showed a markedly increased level of LC3-II compared to RPMI controls. Moreover, when 3MA was added, the LC3-II fraction was decreased (Figure 1), attesting to the validity of the classical autophagy methodologies in human PBMCs.

### Inhibition of autophagy enhances IL-1β production while decreasing TNFα in human PBMCs

**Figure 2:**
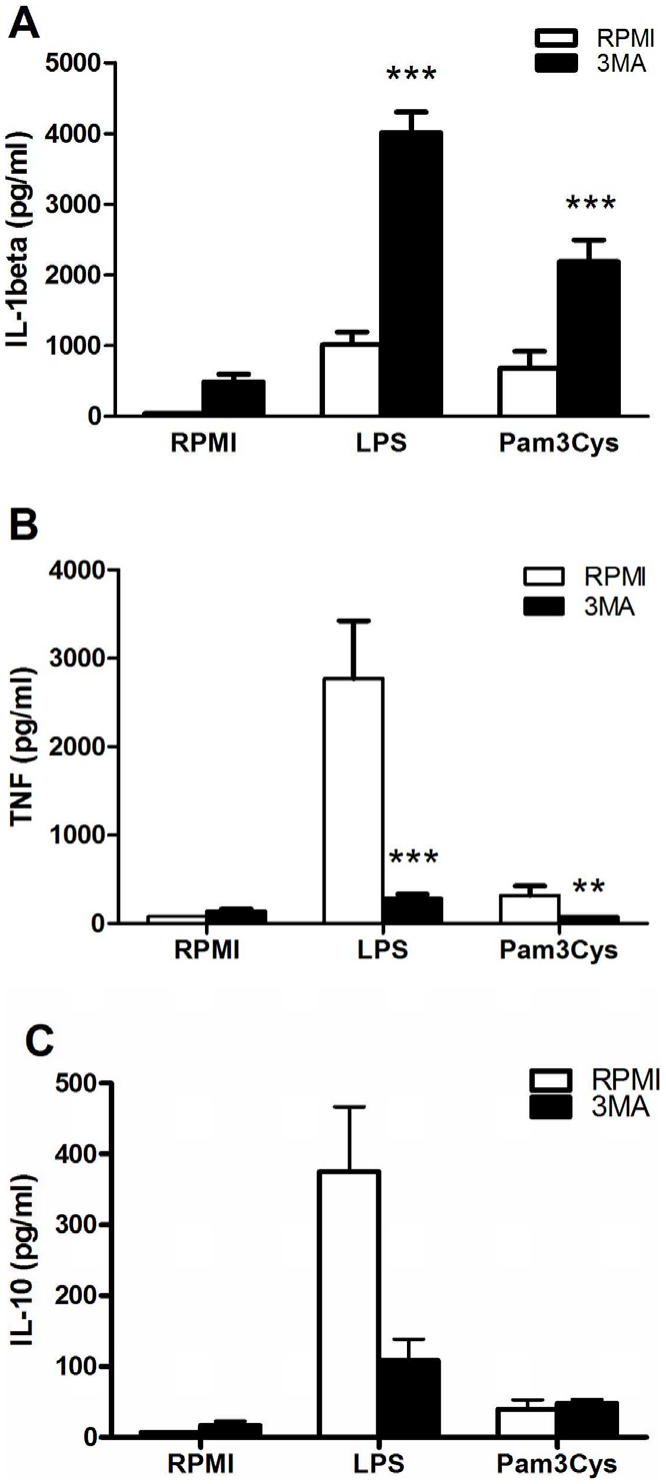
Modulation of inflammatory cytokine production by autophagy inhibition. Freshly isolated human PBMCs were pre-incubated for 1 hour at 37°C in culture medium in the presence or absence of 3MA (10 mM) and afterwards stimulated with culture medium, LPS (10 ng/ml) or Pam3Cys (10 µg/ml). After 24 hours incubation, IL-1β (A), TNFα (B) and IL-10 (C) were measured in the supernatant by specific ELISA. Data are presented as means ± SEM of cells harvested from 15 volunteers, **p<0.01, ***p<0.001.

To assess its effect on the cytokine production, autophagy was inhibited using 3MA, a blocker of the Beclin-1 complex that regulates the initiation of autophagy. PBMCs treated with 3MA showed a significant higher IL-1β secretion after stimulations with TLR2 or TLR4 ligands (Figure 2A). In contrast, TNFα production was diminished in cells conditioned with 3MA compared to controls (Figure 2B). No specific trend for the effect of 3MA on IL-10 production was observed (Figure 2C).

### The modulation of IL-1β and TNFα production is regulated at the transcriptional level

**Figure 3:**
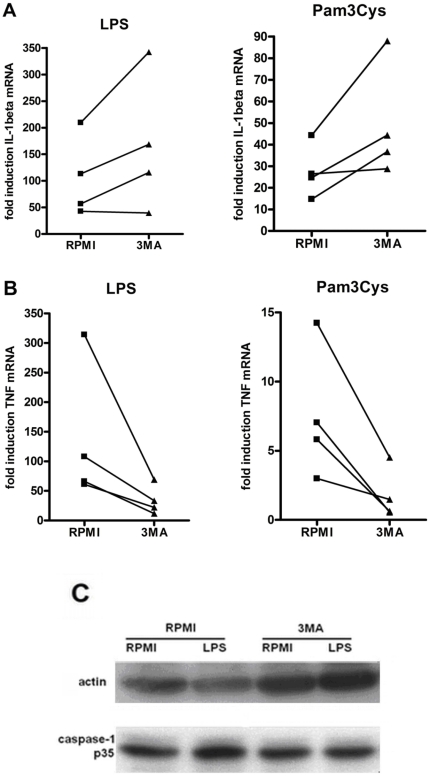
The effects of 3MA on transcription and processing of inflammatory cytokines. Cells pre-treated for 1 hour with culture medium with or without 3MA (10 mM) were stimulated for 4 hours with RPMI, LPS (10 ng/ml) or Pam3Cys (10 µg/ml). RT-PCR was performed and relative levels of IL-1β and TNFα (B) mRNA were determined in 4 volunteers. (C) Western blot of p35 caspase-1 after 1 hour pre-incubation with or without 3MA (10 mM in RPMI), followed by 2 hours stimulation with LPS (10 ng/ml). The picture is representative for results obtained from 6 volunteers.

To identify the level in the cytokine production which is modulated by 3MA, we assessed its effect on transcription and processing of IL-1β. IL-1β mRNA levels were increased in human PBMCs in the presence of 3MA (Figure 3A). Similarly, RT-PCR for TNFα mRNA revealed that the inhibitory effect of 3MA on TNFα production starts with the decrease of the TNFα transcription rate (Figure 3B). On the other hand, western blot analysis of caspase-1 did not show an increased caspase-1 activation (p35 fragment) by 3MA compared to controls (Figure 3C).

### 3MA has an inhibitory effect on the ATP-dependent release of IL-1β

**Figure 4:**
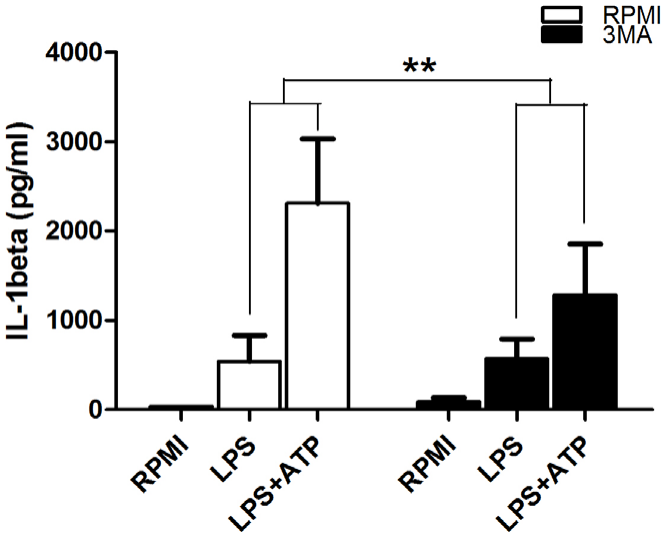
The influence of 3MA on the ATP-dependent release of IL-1β. PBMCs were pre-incubated for 1 hour in the presence or absence of 3MA and were stimulated for 4 hours with LPS (10 ng/ml). After the stimulation, supernatants were discarded and refreshed with RPMI or with RPMI containing 1 mM ATP and cells were incubated for another 15 min. Data are shown as mean ± SEM of supernatant IL-1β levels obtained in 8 volunteers, **p<0.01.

It is known that the inflammasome activator ATP (adenosine triphosphate) is a potent stimulator of IL-1β processing and release [24]. In experiments aiming to assess how this effect is influenced in the circumstances of autophagy inhibition, control PBMCs showed a significantly higher increase (5.73 fold) of secreted IL-1β when exposed to ATP then in samples treated with 3MA (2.25 fold increase) (Figure 4).

### Induction of autophagy by starvation inhibits IL-1β transcription

**Figure 5:**
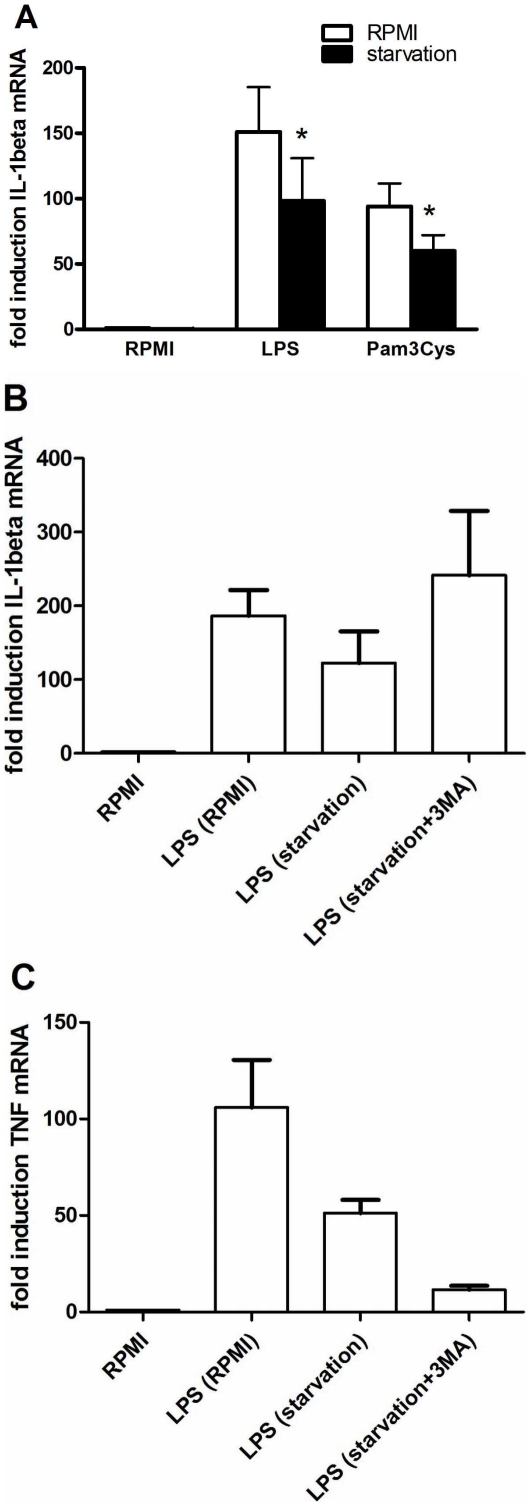
The effects of starvation on mRNA levels of inflammatory cytokines. Cells pre-incubated for 2 hours in either starvation medium (Earle's Balanced Salt Solution) or RPMI were stimulated for 4 hours with medium, LPS (10 ng/ml) or Pam3Cys (10 µg/ml) prepared respectively in starvation medium or RPMI. Subsequently, RT-PCR was performed and IL-1β mRNA levels are depicted as mean ± SEM of cells harvested from 6 volunteers, *p<0.05 (A). RT-PCR results of IL-1β (B) and TNFα (C) mRNA levels in cells pre-incubated for 2 hours with RPMI, starvation medium or starvation medium and 3MA (10 mM) followed by 4 hours stimulation with RPMI or LPS (10 ng/ml). Results are shown as mean ± SEM of data obtained in 4 volunteers.

In an additional set of experiments, we pursued to verify whether consistent findings with those revealed when inhibiting autophagy could be seen in the context of autophagy induction, typically obtained during starvation. In line with the effects observed by the experiments using 3MA, starvation reduced the transcription of IL-1β after LPS or Pam3Cys stimulation of cells (Figure 5A). Furthermore, these effects of starvation were reversed by 3MA, demonstrating the involvement of autophagy in the effects of starvation (Figure 5B). In contrast to its effect on IL-1β, starvation inhibited TNFα mRNA, but 3MA decreased even further TNFα transcription. This observation is consistent with the experiments showed earlier that 3MA reduces the TNFα gene transcription.

### The role of MAP kinases for the modulatory effects of autophagy inhibition by 3MA

**Figure 6:**
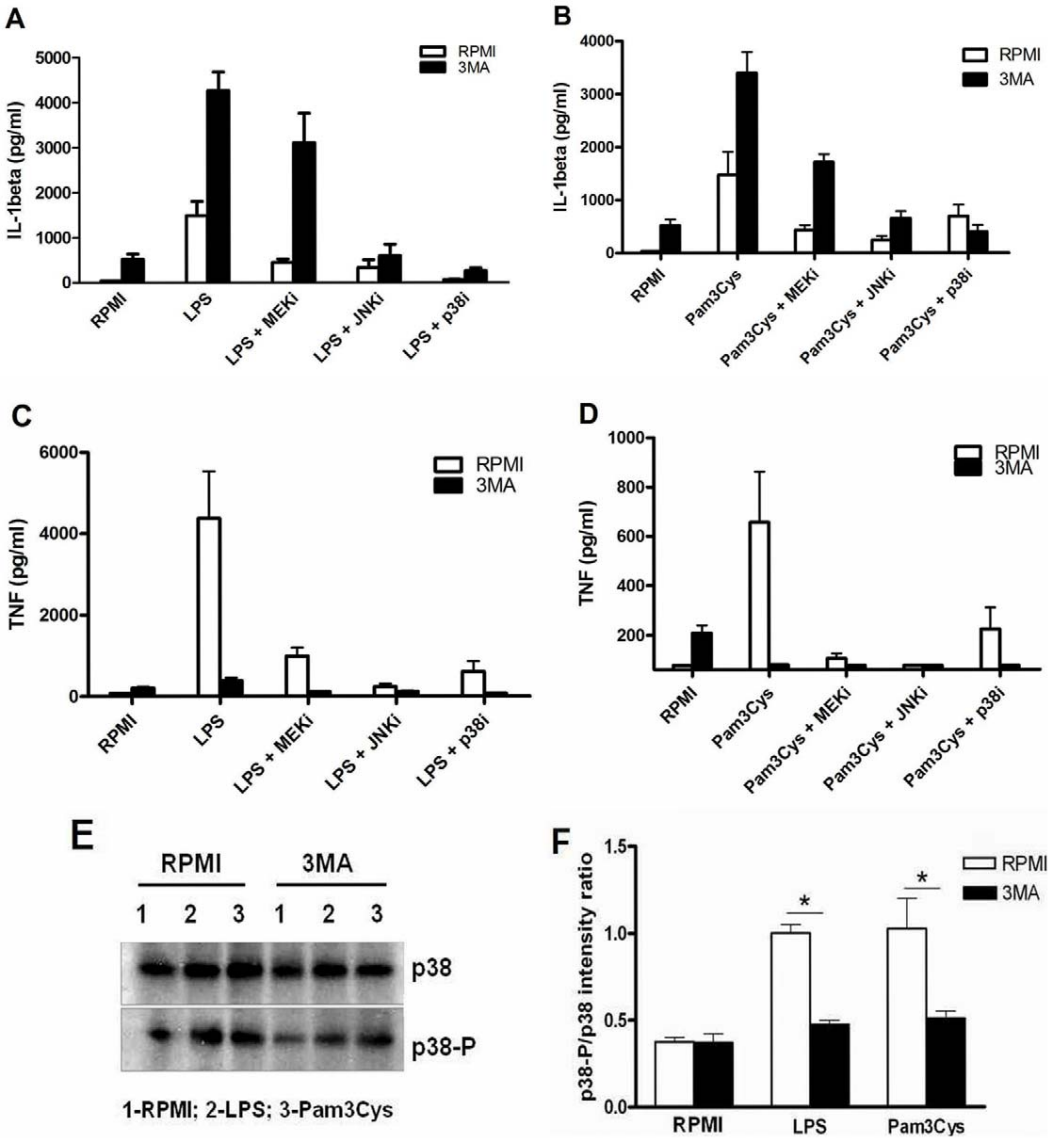
MAPK influence on the 3MA-dependent modulation of IL-1β and TNFα production. PBMC samples conditioned for 1 hour in either medium or in the presence of MAPK inhibitors: MEK inhibitor (5 µM), JNK inhibitor (20 µM) or p38 inhibitor (1 µM) were subjected to 1 hour treatment with RPMI or 3MA (10 mM), followed by stimulation with RPMI, LPS (10 ng/ml) or Pam3Cys (10 µg/ml). After 24 hours incubation, specific ELISA was performed to determine the level of IL-1β in response to LPS (A) and Pam3Cys (B) stimulations, same as for TNFα (panels C and D, respectively). Data from 6 volunteers are shown as mean ± SEM. (E) Western blot of total and phosphorylated p38 in cells pre-treated for 1 hour with RPMI or 3MA (10 mM) and subjected to 1 hour stimulation with RPMI, LPS (10 ng/ml) or Pam3Cys (10 µg/ml). (F) Quantification of the effect of 3MA on the phosphorylation of p38.

Mitogen-activated protein kinases (MAPKs) such as ERK1/2 (activated by MEK), JNK or p38 are important intracellular mediators of the stimulation of pro-inflammatory cytokine production during innate immune responses [25]. In order to decipher the mechanism through which autophagy inhibition modulates cytokine transcription, we investigated the effects of MAPK inhibition on the 3MA-dependent variation of cytokine production. Cells pre-treated with inhibitors of MEK, JNK or p38 in the presence or absence of 3MA have further been stimulated with LPS and Pam3Cys and cytokine production is depicted in Figure 6 (panels A and B for IL-1β; C and D for TNFα). With all inhibitors, except p38i, 3MA had similar consequences on the cytokine production, consisting in increasing IL-1β and decreasing TNFα secretion after both LPS and Pam3Cys stimulation. Nevertheless, when using p38i, lower IL-1β concentrations were measured in samples treated with 3MA after Pam3Cys stimulation than in controls. Consequently, in order to test the hypothesis of p38 being involved in the effects of 3MA, we performed Western blots of total and phosphorylated (active) p38 which revealed that in samples treated with the autophagy inhibitor, the phosphorylated fraction of p38 is lower than in controls, after TLR4 or TLR2 stimulations (Figure 6E). Furthermore, the slight induction of TNFα and IL-1β by 3MA alone was shown to be dependent on p38 MAPK signaling (3MA: IL-1β 665±135 pg/ml, TNFα 215±45 pg/ml and 3MA+p38i: IL-1β 20±10 pg/ml, TNFα 40±15 pg/ml; p<0.05 for both IL-1β and TNFα).

## Discussion

Earlier studies linked autophagy to innate immunity as a defense system against invading pathogens in nonphagocytic cells [26]. Later it has been also demonstrated that autophagy regulates pathogen-associated molecular pattern (PAMP) recognition by facilitating TLR stimulation in response to viral antigens [27]. Subsequently, it has been discovered that components of the autophagy machinery are used for intracellular degradation of bacteria and MHC-II dependent activation of specific immunity [9]. These effects could prove very relevant, as genetic polymorphisms of the autophagy genes *ATG16L1* and *IRGM* are associated with Crohn's disease [15]–[17]. In addition, deletion of the autophagy gene *ATG16L1* in mice resulted in inflammasome activation and more severe experimental colitis [22]. However, the effect of autophagy on IL-1β production was demonstrated only in murine cells, and no information was available whether similar effects may be exerted in human cells.

In the present study, using a complementary approach by both inhibition and induction of autophagy as verified using the LC3-II marker, we show that autophagy has strong inhibitory effects on IL-1β production. These results add to the increasing body of evidence showing that basal autophagy in physiological conditions has important modulatory effects on inflammation [22], [28]. However, in contrast to murine cells, autophagy did not inhibit inflammasome activation in human PBMCs, and its effects were the consequence of the modulatory effect on transcription of cytokine genes. These data point out once again that inflammasome activation and regulation differs greatly in human PBMCs and murine macrophages, as inflammasome activation is constitutive in human monocytes, but not in mouse macrophages [29]. Subsequently, in human PBMCs, caspase-1 activation is not increased by autophagy inhibition. In addition, the assessment of ATP/inflammasome-dependent release of IL-1β shows that this is not increased in the presence of 3MA compared to controls. This would suggest that although the cytokine release appears to be slightly inhibited on the short term (15 minutes after ATP addition), transcriptional regulation is sufficient to determine the higher levels obtained in longer stimulations. These findings confirm that the increase of IL-1β production during autophagy inhibition is determined by the activation of transcription and is not due to the influence on the subsequent steps of processing by caspase-1 or secretion from the intracellular compartment.

In the attempt to point out to the mechanism underlying the effect of autophagy on gene transcription, we have tested the importance of several MAPK-dependent mechanisms and observed that the only condition that reversed the stimulatory effects of 3MA was the p38 inhibitor SB202190. As there is evidence of this inhibitor also influencing Rip2 activation [30], the observed effect might also be involving other p38-related MAP kinases in addition to p38. However, while these results have led us to the hypothesis that p38 activation might be important for the effect of 3MA on IL-1β, this was not confirmed by Western blot analysis, as phosphorylated p38 (active form) was decreased in 3MA treated samples. However, in line with earlier evidence showing that p38 phosphorylation is positively linked to the process of autophagy [31], [32], 3MA treatment led to inhibition of p38 phosphorylation and this could, at least in part, represent a pathway of autophagy effect on TNFα release. In summary, while the inhibitory effects of 3MA on p38 phosphorylation can explain its effect on TNFα production, this cannot be the cause of the potentiation of IL-1β transcription, and further studies are needed to decipher this aspect.

Recently, two new studies have also reported that the peptidoglycan receptor NOD2, whose gene is considered a susceptibility locus for Crohn's disease, is linked to autophagy by recruiting ATG16L1 at the site of the microbial entry within the infected cell [21], directing microbial engulfment in the autophagosome and inducing antigen presentation [20]. These latest two studies represent the connection between the two genetic pathways that induce susceptibility to Crohn's disease: NOD2 and autophagy. Crohn's disease is a chronic inflammatory reaction in the gut mucosa, and recent studies strongly suggested a role of autophagy in this process. On the one hand, autophagy seems to be crucial for the homeostasis of Paneth cells. Mice with a defective ATG16L1 display abnormal Paneth cells with a lower amount of granules containing antibacterial defensins, while in turn displaying an increased IL-1β production [28]. These effects could lead to bacterial persistence in the mucosa and overwhelming inflammation. The studies by Cooney *et al.* [20] and Travassos *et al.* [21] show for the first time that a decreased autophagy in humans can lead to decreased bacterial clearance, while our study provides evidence that diminished autophagy in human cells (e.g. through pharmacological inhibition as shown here, or through genetic mutations in Crohn's disease) could lead to uncontrolled IL-1β production.

The differential findings in mice and humans concerning the modulation of IL-1β by autophagy, transcription in humans and caspase-1 activation in mice, remains to be elucidated. One possible explanation lies in the different cell populations studied: monocytes in our study in humans versus macrophages in the mouse studies. We have previously shown clear differential regulation of the caspase-1 inflammasome in monocytes (constitutive activation) versus macrophages (inducible activation) [29]. Another source of difference could be species specificity, an aspect which in terms of the autophagy-inflammation interaction is still largely unknown. This is an aspect which certainly deserves attention in future studies, and the present study represents an important first step in this direction.

In conclusion, this study is the first attempt to investigate the modulation of inflammatory cytokines by the process of autophagy in humans. We have shown that disruption of autophagy has an important impact on inflammatory cytokine modulation, determining a remarkable and specific increase of IL-1β secretion, while decreasing TNFα production. We demonstrate that the inflammasome activation is not influenced in human PBMCs and that the changes are exerted at the gene transcription level. Although the p38 MAPK pathway seems to be linked to the autophagy process and our findings may explain the effects on TNFα production through modulation of p38 phosphorylation, this is probably not the mechanism of the observed higher IL-1β mRNA levels in the circumstances of autophagy inhibition. Further assessment of the mechanisms through which inflammation is regulated by autophagy may bring new opportunities for the understanding and treatment of autoinflammatory disorders such as Crohn's disease.

## Materials and Methods

### Reagents

3-Methyl Adenine (3MA) was purchased from Sigma (St. Louis, MO). LPS (*E. coli* serotype 055:B5) was purchased from Sigma. Synthetic Pam3Cys was purchased from EMC Microcollections (Tubingen, Germany). Anti-actin (A2066) antibody was purchased from Sigma; anti-human caspase-1 p10 (sc515) antibody was purchased from Santa Cruz Biotechnologies (Santa Cruz, CA); anti-human/mouse LC3B antibody (NB600-1384) was purchased from Novus Biologicals (Cambridge, UK); anti-human total p38 antibody (CS9212) and phosphorylated p38 antibody (Phospho p38 MAPK Thr180/Tyr182 Antibody, CS4511) were purchased from Cell Signaling (Danvers, MA). oxATP was purchased from Sigma. SB202190 p38/Rip2 MAPK inhibitor (p38i); SP600125, JNK1/2/3 inhibitor (JNKi) and U0126 MEK1/2 inhibitor (MEKi) were purchased from Superarray Bioscience Corporation (Bethesda, MD). In experiments using pharmacological inhibitors, control cells were treated with an equivalent concentration of vehicle (0.1% DMSO).

### PBMC isolation and stimulation

Peripheral blood was harvested from the antecubital vein of healthy volunteers, after obtaining informed consent. The PBMC fraction was obtained by differential centrifugation over Ficoll-Paque (Sigma). Cells adjusted to 5×10^6^ cells/ml were suspended in culture medium RPMI (Roswell Park Memorial Institute) 1640, supplemented with 50 µg/ml gentamicin, 2 mM L-glutamine and 1 mM pyruvate. All cytokine induction experiments were performed in duplicate wells. Cells were pre-incubated for 1 hour at 37°C in culture medium or in the presence of 3MA (10 mM). After the pre-treatment, RPMI (negative controls), purified LPS (10 ng/ml) or Pam3Cys (10 µg/ml) was added to the cells. In separate experiments, cells were first incubated for 1 h with RPMI, MEK inhibitor (5 µM), JNK inhibitor (20 µM) or p38 inhibitor (1 µM) before performing the steps described above. After 24 hours, the supernatants were collected and stored at −20°C until assayed.

To investigate the effect of 3MA on the ATP-dependent IL-1β release, cells pre-treated for 1 hour with RPMI with or without 10 mM 3MA were stimulated for 4 hours with LPS (10 ng/ml or 1 µg/ml). After the stimulation, supernatants were discarded and refreshed with RPMI containing 1mM ATP, after which the cells were incubated for another 15 min. The LPS-dependent IL-1β production during the first 4 hours, and the ATP-dependent IL-1β secretion after the additional 15 minutes, was assessed in the supernatant.

Autophagy induction was performed in adherent monocytes using starvation medium, Earle's Balanced Salt Solution, EBSS (Invitrogen, Carlsbad, California). PBMCs were incubated for 1 hour after which the supernatant containing the non-adherent lymphocytes was discarded. Adherent cells were pre-treated for 2 hours with starvation medium. Subsequently, cells were stimulated for 4 hours with LPS or Pam3Cys prepared in starvation medium. In parallel, controls were given the same treatment using RPMI. After 4 hours, supernatants were discarded and TRIzol Reagent was added to the cells which were subsequently frozen and stored at −80°C until assayed. In different experiments, 3MA was also used in combination with starvation medium to investigate whether the effects of starvation are reversed by 3MA.

Both experiments involving stimulation or inhibition of autophagy were performed with cells isolated from the same healthy volunteers. However, in some experiments in which not all experiments could be performed with blood collected from the same volunteers, studies were done in cells collected from additional healthy volunteers.

### Cytokine measurements

Cytokine concentrations were determined using specific sandwich ELISA kits for IL-1β, TNFα (R&D Systems), and IL-10 (Sanquin).

### RT-PCR

Samples stimulated for 4 hours at 37°C were treated with TRIzol Reagent (Invitrogen) and total RNA purification was performed according to manufacturer's instructions. Isolated RNA was subsequently transcribed into complementary DNA using iScript cDNA Synthesis Kit (Bio-Rad) followed by quantitative PCR using the Sybr Green method. The following primers were used in the reaction: IL-1β forward 5′-GCCCTAAACAGATGAAGTGCTC-3′ and reverse 5′-GAACCAGCATCTTCCTCAG-3′, TNFα forward 5′-TGGCCCAGGCAGTCAGA-3′ and reverse 5′-GGTTTGCTACAACATGGGCTACA-3′, β2-microglobulin forward 5′-ATGAGTATGCCTGCCGTGTG-3′ and reverse 5′-CCAAATGCGGCATCTTCAAAC-3′ (Biolegio). Results are shown as fold increases in mRNA levels in stimulated samples compared to controls. Quantitative PCR for cytokines was used especially in short-term induction of autophagy experiments using starvation medium, in which long-term incubation led to a high percentage of cell death, and in which ELISA cytokine measurements in the supernatants were not able to provide an appropriate assessment of cytokine stimulation.

### Western blot

For western blotting of actin, caspase-1 and p38 MAPK (total and phosphorylated), 5×10^6^ cells were lysed in 100 µl lysis buffer (50 mM Tris, pH 7.4, 150 mM NaCl, 2 mM EDTA, 2 mM EGTA, 10% glycerol, 1% Triton X-100, 40 mM β-glycerophosphate, 50 mM sodium fluoride, 200 µM sodium orthovanadate, complete mini EDTA-free protease inhibitor cocktail (Roche) and PhosSTOP Phosphatase Inhibitor Cocktail (Roche)). The homogenate was stored at −20°C. When needed, samples were thawed, then centrifuged for 10 min at 14,000 rpm, and the supernatant was taken for western blotting. Equal amounts of protein were subjected to SDS-PAGE electrophoresis using 12% polyacrylamide gels at a voltage of 100V. After separation, proteins were transferred to polyvinylidene fluoride (PVDF) membrane using the dry blotting method (iBlot™, Invitrogen). The membrane was blocked with 5% (w/v) milk powder in Tris-buffered saline/Tween 20 (TBST) for 1 hour at room temperature followed by incubation over night at 4°C with the primary antibody 1∶500 in 5% (w/v) BSA/TBST (5% bovine serum albumin/TBST). After overnight incubation the blots were washed three times with TBST and incubated with HRP-conjugated anti-rabbit antibody at a dilution of 1∶5000 in 5% (w/v) milk powder in TBST for 1 hour at room temperature. After washing three times with TBST the blots were developed using ECL Plus Western Blot Detection Reagents (Amersham Biosciences, Buckinghamshire, UK). For western blotting of LC3, an amount of 10×10^6^ cells were lysed after being cultured in media containing the inhibitors of lysosomal fusion, ammonium chloride,20 mM, and leupeptine,100 µM. After protein electrophoresis in 15% polyacrylamide gel, the transfer was performed on nitroglycerine membranes using the wet blotting method (Bio-Rad) and was followed by blocking, incubation with first and then second antibody, each time using 5% (w/v) milk powder in Tris-buffered saline/Tween 20 (TBS-T). After washing 3 times with TBS-T, blots were developed using the Super Signal® West Femto Maximum Sensitivity Substrate (Thermo Scientific, Rockford, IL, USA) according to the manufacturer's instructions. Quantitative assessment of band intensity was performed by Image Lab statistical software (Bio-Rad, CA, USA).

### Statistical analysis

The differences were analysed using Wilcoxon signed rank test and were considered statistically significant at a p-value<0.05. Data are shown as cumulative results of levels obtained in all volunteers (means ± SEM).
